# Sensitive Dentine

**Published:** 1860-09

**Authors:** Geo. F. Foote


### SENSITIVE DENTINE.

BY GEO. F. FOOTE, M. D.

FROM the days of Hippocrates to the present time, the honorable profession of medicine has passed, and is even now passing, through successive changes. The laws of cure are viewed from different stand points, and the means used to acquire the desired ends vary with each succeeding generation, as each with a progressive spirit deems itself wiser than its predecessors.

This desire for change and advancement is found in all departments of civilized life.

And in this respect, what has been the history of medicine and other sciences, will doubtlessly be the history of dentistry.

Indeed we already see changes, and with thankful hearts welcome the new light as it daily flows in upon us. And may it grow brighter and clearer as we grow older and wiser. And while watching for this light, let us do it with a helping hand, acting well our part; developing and radiating truths fraught with blessings to mankind that shall pierce the circumference of our individual existence and tell to the world that we, too, have performed a use while in it.

As we look over some of the ancient works upon medicine, written by the older savans, we find in them many singular, and in appearance to us, very absurd prescriptions for the sick. So ridiculous do they appear, that one can not help but smile as he reads, at their crudeness and apparent folly.

Query—will the future look back to this time and find similar and equally as crude absurdities in the present profession of dentistry? Are the writings of our wise men, gifted with all the lore found in modern dentistry, to be laid upon the dusty shelves of the future profession, and only kept as literary curiosities, to excite the ridiculous in those who revel in ancient dental literature? It would be a noble work and a pleasant duty, could we but lift the cloud from, and unveil to ourselves our own errors and short comings in practice, a knowledge of which would lead to valuable improvements, that might subserve present uses and anticipate the future.

We are led to these reflections by a careful study of what is written and taught in regard to the various affections of the teeth and the treatment therefor; particularly that of Sensitive Dentine.

Whenever by decay or accident the enamel is removed from a portion of the tooth bone, the tubuli which serve as mediums of sensation are ruptured and exposed to the atmosphere and other irritants of the mouth. By reason of which these often take on augmented sensibility, become painful to the touch, and by common consent we call it *Sensitive Dentine.* Healthy dentine is not devoid of sensibility, but it is sensitive only to a certain extent, and can be incised with but little suffering. Not so with that which has become irritated from exposure. This is exceedingly painful to the most delicate manipulations; that part being most so where the anastimosing or terminal branches of the tubuli are most numerous, viz: at the juncture of the enamel and tooth bone.

But sensitiveness does not always follow decay. And in such teeth the tubuli become solidified in advance of the decomposition, hence are incapable of conveying nervous sensation. And here is one of the beautiful results growing out of the efforts of the organism to protect and preserve, by proper safeguards her delicate and important structures, against threatened violence from external causes.

The presence of a foreign body stimulates the vital forces to produce this change. Hence we see in teeth where the decay has been general, this change or substitution of cementum, or if you please, this ossification extending to the obliteration of the entire pulp; somewhat analogous to the formation of pearls in oysters, the growth of which is excited by the presence of a grain of sand.

The same results are induced by a proper filling. The tubuli immediately surrounding it being in time solidified.

This fully explains to us why teeth that are irritated by the changes of temperature through the medium of a metallic filling in time lose this augmented sensibility.

With these facts before us, what are the indications when from sensitive dentine the necessary manipulations to secure a proper filling are too painful? or when the cavity of decay is so large that there remains but a thin lamina of dentine covering the pulp, so thin that the presence of a metallic filling, from its highly conductive power, may, with the changes of temperature and electrical influences induce inflammation and suppuration? Or when a healthy pulp or a stray branch from it is slightly wounded with some hemorrhage? What is the practice recommended through our dental journals and conventions, and in common use among dentists? What is "*the treatment*" that every tyro in practice talks so much about? What, but the application of an irritant, escharotic or some destructive agent.

Some apply arsenious acid, some cobalt, some tannic acid, some nitrate of silver, some chloride of zinc, some nitric acid, and nearly all creosote.

And what are the consequences? Exactly the same as when similar applications are made to the abraded surfaces of other parts of the body, viz: inflammation and its sequences, more or less suffering and frequent loss of the pulp and sometimes of the tooth.

Now to us this crude way of doctoring so important an organ as a tooth seems all wrong. Our reason does not assent to it. Our practical experience does not convince us that there is any philosophy in it or good results following it. The discouraging reports to the dental associations of those who from their ill success have abandoned the saving of a living pulp, that has been in the least exposed, or that has but a slight covering of dentine over it, confirm us in these opinions.

To make our prognosis favorable we must, so far as we have the means of doing it, allay all irritation and avoid as much as possible all causes producing it. The parts we have to deal with are most delicate in their organization and as susceptible to external influences when exposed as any of the soft tissues not incased in bone.

Are we then to allay or prevent irritation by the application of agents that chemically destroys the vitality of the tissues brought in contact with them? Does this not excite an inflammation beyond the parts destroyed, or rather is it not a necessary sequence from the efforts of the system to throw or slough off the effete matter caused by these escharotics? What does the surgeon do when the surface of the body is denuded by an incision or by a removal of any portion of its natural covering. He excludes the air by some artificial contrivance, brings the cut surfaces in apposition and leaves the rest to the *vis medicatrix natura.* What does simple nature unassisted by the surgeon do? She covers the exposed surfaces with a crust or scab and thus excludes the air and other influences while the internal changes are going on for the restoration of the parts.

All these point out to the dentist the rational treatment of sensitive teeth, which consists in sealing up hermetrically the cavity of exposure to the exclusion of all irritating influences with some soft filling until through the vital forces such physical changes are made as shall render them insensible to the manipulations necessary to a proper filling and to the presence of gold.

That these changes will occur resulting in a healthy condition of the tooth, we are fully confident of, from our own experience and observation.

This, if you please, may be called the expectant treatment, and *cœterus paribus* will not be found wanting in its results, except in some vitiated states of the organism where there is a deficiency in the recuperative powers.

In conclusion, then, should we in preparing a cavity find our patient suffering much from sensitive tooth bone, we would remove as much of the decay only, as could be done without giving pain, and after drying out the cavity hermetrically seal it up with some of the preparations of gutta percha that are non-medicinal. Should the cavity be dishing or too shallow to retain a filling we would use gutta percha made adhesive by the addition of rosin or gum elemi.

In from six to ten days we should expect to be able to excavate and refill the same cavity, to our own entire satisfaction without suffering to the patient. But the time necessary for this change may vary in different patients, depending upon the quality of the teeth, the age and the condition of the system. Should we find the parts sensitive from a close proximity to the pulp cavity, or should we accidently wound a stray branch from a healthy pulp so as to induce hemorrhage we should pursue the same treatment with the addition of a longer interval of time between the temporary and permanent fillings, say from two to six months.

Thus would we treat them; abjuring the direct application of all medicaments or stimulents of any kind whatever. It becomes then simply a question of time and patience which are known as sovereign remedies to many of the ills that flesh is heir to.

